# Development of a Digital Application Program Based on an Institutional Algorithm Sustaining the Decisional Process for Breast Reconstruction in Patients with Large and Ptotic Breasts: A Pilot Study

**DOI:** 10.3390/cancers17111807

**Published:** 2025-05-28

**Authors:** Federico Ziani, Andrea Pasteris, Chiara Capruzzi, Emilio Trignano, Silvia Rampazzo, Martin Iurilli, Corrado Rubino

**Affiliations:** 1 Department of Medicine, Surgery and Pharmacy, University of Sassari, 07100 Sassari, Italy; federico.ziani@aouss.it (F.Z.); etrignano@uniss.it (E.T.); corubino@uniss.it (C.R.); 2 Plastic Surgery Unit, University Hospital Trust of Sassari, 07100 Sassari, Italy; 3 Plastic, Reconstructive and Aesthetic Surgery Training Program, University of Sassari, Viale S. Pietro 43, 07100 Sassari, Italy; 4 Department of Plastic and Reconstructive Surgery, University of Trieste, 34127 Trieste, Italy; martin.iurilli@phd.units.it

**Keywords:** clinical decision support system (CDSS), immediate breast reconstruction, reconstructive algorithm, mammoplasty, mastectomy

## Abstract

Background/Objectives: Immediate implant-based breast reconstruction is an established option for selected patients undergoing mastectomy. However, patients with large and ptotic breasts present specific reconstructive challenges, often requiring tailored approaches to minimize complications and optimize aesthetics. This pilot study aimed to evaluate the clinical feasibility and effectiveness of a mobile application developed to support intraoperative decision-making based on an institutional algorithm for breast reconstruction. It is also important to underline that this pilot study was exploratory in nature and primarily aimed at assessing feasibility and adherence to an app-based decision pathway, rather than comparative efficacy. Methods: We conducted a prospective observational study from October 2023 to December 2024 at the University Hospital of Sassari. Female patients with large and ptotic breasts undergoing immediate implant-based reconstruction were included. A mobile app, developed using MIT App Inventor 2, implemented our institution’s algorithm and guided surgeons through both preoperative and intraoperative decision-making. Surgical options included subpectoral, prepectoral with autologous fascial flaps, or prepectoral with acellular dermal matrix (ADM) reconstruction, depending on flap thickness and fascia integrity. Results: Sixteen patients (21 reconstructed breasts) were included. Surgical planning and execution followed app-generated recommendations in all cases, with no intraoperative deviations. Subpectoral reconstruction was performed in six patients, prepectoral with ADM in eight, and prepectoral with fascial flaps in two. The app was rated positively by all surgeons and facilitated consistent decision-making. Conclusions: The proposed mobile application, described in this pilot study, proved to be a feasible and effective decision-support tool for implant-based breast reconstruction in patients with challenging anatomy. It standardized surgical choices, supported training, and has the potential to enhance reproducibility and safety in complex reconstructive procedures.

## Simple Summary

Immediate breast reconstruction after mastectomy helps improve patients’ quality of life, especially in women with breast cancer who require surgery. However, reconstruction in patients with large and sagging breasts is often more complex due to anatomical challenges and the risk of complications. To help plastic surgeons make faster and more accurate choices during surgery, our team developed a mobile application that follows a step-by-step decision-making pathway. The app is based on a previously validated surgical algorithm and guides the user in selecting the most appropriate reconstructive option, depending on the patient’s anatomy and surgical findings. In this pilot study, we evaluated the app in clinical practice and found that it supported consistent decisions across different surgeons and was well accepted by both senior staff and trainees. This tool could help standardize care, reduce variability, and improve outcomes in complex breast reconstructions.

## 1. Introduction

The evolution of breast cancer surgery has shifted toward treatment paradigms that prioritize not only oncologic safety but also patient-centred outcomes. Immediate breast reconstruction following mastectomy is now recognized as a standard option in comprehensive breast cancer care, contributing significantly to improved body image, psychological well-being, and overall quality of life, particularly when performed in a one-stage [1], direct-to-implant approach. However, reconstructive strategies in patients with large and ptotic breasts remain complex due to anatomical variability, skin redundancy, and the elevated risk of postoperative complications such as flap necrosis and implant exposure [2,3]. This is particularly relevant in patients with macromastia, where skin-reducing mastectomy techniques have demonstrated favorable outcomes [4].

To address these challenges, several surgical algorithms and protocols have been developed [5,6]. Among them, our institution previously introduced a decision-making algorithm based on the intraoperative evaluation of skin flap thickness and the integrity of the pectoral fascia, guiding the surgeon through appropriate choices between subpectoral reconstruction, pre-pectoral reconstruction with autologous fascial flaps, or acellular dermal matrices (ADMs) [7]. Despite its clinical validity, the application of such algorithms has traditionally relied on subjective interpretation and surgeon experience, which may introduce variability in surgical decision-making.

In recent years, the integration of digital technology into surgical practice has opened new frontiers in real-time, standardized decision support. Mobile applications, in particular, are emerging as powerful tools capable of enhancing intraoperative decision-making, promoting consistency across surgical teams and serving as valuable educational resources for trainees [8,9,10]. These tools enhance patient engagement and recovery, but their widespread implementation remains challenged by integration barriers and the need for clinical validation across surgical workflows [11,12]. While validated models for their use in plastic and reconstructive surgery remain scarce, digital tools have already been widely adopted in other perioperative phases, especially for postoperative care and patient monitoring, where technologies such as mobile apps and connected devices support communication, complication detection, and recovery tracking [13,14].

To bridge this gap, we developed a mobile application that implements our institutional algorithm for immediate implant-based breast reconstruction in patients with large and ptotic breasts. The app guides surgeons through a structured binary flowchart based on objective clinical parameters, allowing standardized decisions in real time. By reducing inter-operator variability, the app not only improves the reproducibility of reconstructive planning but also may represent a scalable tool for surgical education and multidisciplinary alignment. In recent years, mobile health (mHealth) technologies, such as smartphone applications, wearable sensors, and digital questionnaires, have become increasingly common in surgical practice. These tools are being used to remotely monitor postoperative recovery, evaluate physical fitness, and collect patient-reported data throughout the perioperative period. While these solutions have the potential to improve patient engagement and recovery, their integration into everyday surgical workflows still presents some practical and organizational challenges, and further research is needed to evaluate their long-term impact [11,13]. Some digital interventions have shown positive results when adapted to specific surgical pathways and developed with active patient involvement, particularly in supporting preoperative psychological preparation and behavior change [15], while others have been more focused on encouraging healthy lifestyle habits and self-management after surgery [16].

This pilot study reports the clinical results of the first prospective series of patients treated using the app-based algorithm. We evaluate its feasibility, adherence to the recommended reconstructive pathways, and associated clinical outcomes. Ultimately, our goal is to assess how technological innovation can optimize surgical workflows and improve both clinical and educational standards in breast reconstruction.

## 2. Materials and Methods

### 2.1. Study Overview

This prospective observational study was conducted between October 2023 and December 2024 at the Plastic Surgery Unit of the University Hospital of Sassari, Italy. The primary aim was to evaluate the clinical applicability and effectiveness of a mobile application developed to implement a previously validated institutional algorithm for immediate implant-based breast reconstruction in patients with large and ptotic breasts [7].

The surgical team included 3 senior consultants (>10 years experience) and 2 residents in their final years of training, all of whom participated in app-based decision-making.

All surgeons in our unit were invited to complete anonymous postoperative questionnaires assessing the app’s usability, clarity, and clinical utility.

### 2.2. Institutional Algorithm Integration

The institutional algorithm, originally described by Rampazzo et al., includes two distinct but integrated phases:

- Preoperative evaluation, aimed at selecting the most appropriate skin excision pattern based on tumour location, oncologic requirements, and the presence of prior surgical scars. This step distinguishes between a Wise pattern (Type IV) and a Modified Wise pattern (Type V) [17,18] approach, ensuring optimal management of skin redundancy and symmetry.
- Intraoperative evaluation, focused on assessing the mastectomy flap thickness [19] and pectoralis fascia integrity, which are critical to selecting one of three reconstructive options:
  - Subpectoral reconstruction;
  - Prepectoral reconstruction with fascial flaps;
  - Prepectoral reconstruction with ADM.

Both decision points are implemented and supported in the digital application used in this study.

### 2.3. App Design and Functionality

**Figure 1:**
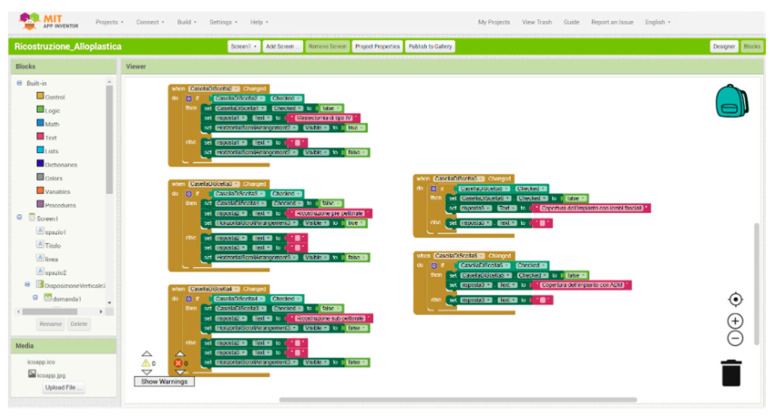
Schematic overview of the mobile app interface and logic flow. Each decision point corresponds to a binary clinical parameter (e.g., flap thickness, fascia integrity) that directs the user to the most suitable reconstructive option (subpectoral, prepectoral with ADM, or prepectoral with fascial flaps).

**Figure 2:**
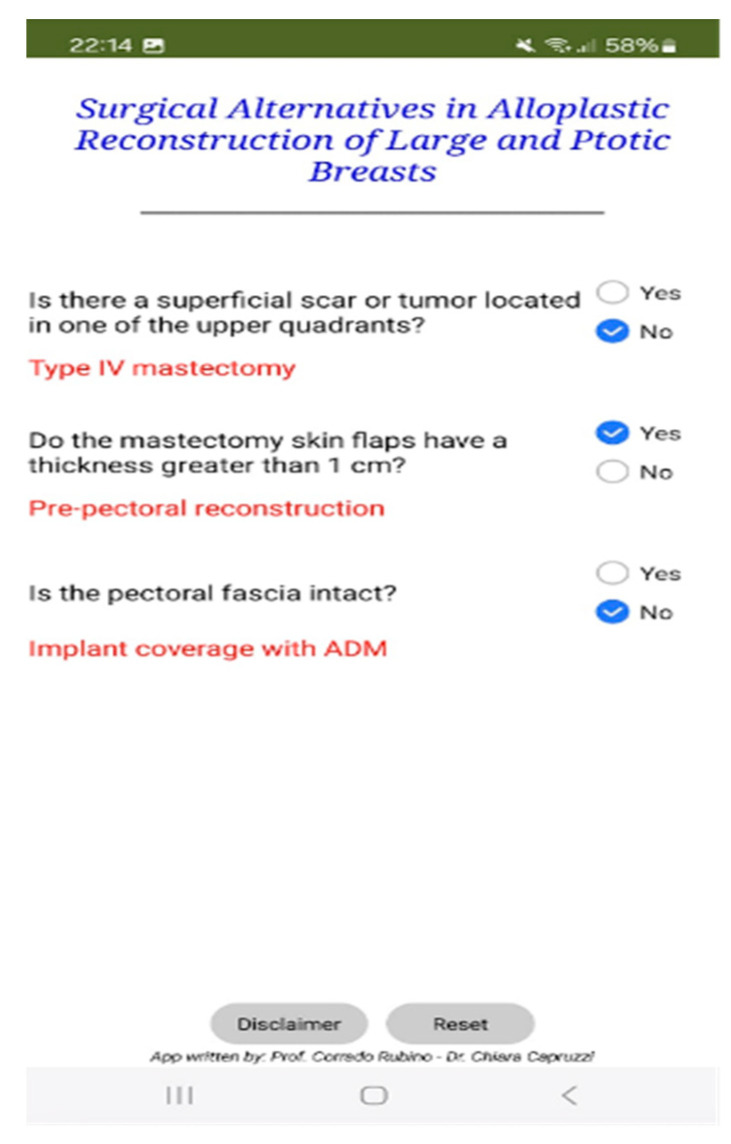
Application interface.

The mobile application was developed using MIT App Inventor 2, an open-source platform that enables development through a visual, block-based programming environment (Figure 1 and Figure 2). This architecture simplifies the implementation of decision-making algorithms, particularly in clinical settings, by facilitating a logic flow that is both intuitive to visualize and easy to modify.

The app’s structure follows a sequential block design, where each user input leads to a predefined output, effectively reflecting the underlying decision-tree logic of the institutional algorithm.

The application features a user-friendly graphical interface and mirrors the stepwise reasoning of the algorithm by guiding users through:

- Preoperative decision-making, based on binary inputs regarding tumour location, required skin excision, and the presence of scars, leading to the selection of Wise IV or Wise V skin patterns.
- Intraoperative decision-making, based on real-time clinical evaluation of flap thickness (measured via palpation and ruler) and fascia integrity, culminating in automated recommendations for reconstruction type.

A disclaimer page precedes app usage, summarizing inclusion and exclusion criteria, and clarifying that the tool is intended to support—not replace—clinical judgment.

### 2.4. Inclusion Criteria

Female patients were eligible if they had large and ptotic breasts, defined by:

- Nipple-to-sternal notch distance ≥ 26 cm;
- Nipple-to-inframammary fold (IMF) distance ≥ 8 cm.

Patients with prior radiotherapy, BMI > 40, collagen vascular diseases, vasculitis, inflammatory breast cancer, or smoking > 20 cigarettes/day were excluded.

### 2.5. Clinical Workflow and Implementation

All included patients underwent preoperative planning using the app, which helped determine the skin pattern (Type IV or Type V). During surgery, the app was again employed for intraoperative decisions regarding the reconstructive technique.

Surgeons followed app recommendations in all cases. Algorithm adherence was documented intraoperatively by an assistant not involved in the surgical procedure. All decisions made through the app were compared to the procedure executed and recorded prospectively. The app facilitated the real-time standardization of the decision-making process among all surgical operators, with no intraoperative protocol deviations reported. Additionally, the app was rated as a practical support tool by both attending surgeons and trainees, reinforcing its value for both clinical and educational use.

## 3. Results

A total of 16 patients were included in the study, with a mean age of 55.4 years (range 47–68) and a mean BMI of 28.3 kg/m^2^ (range 19.7–38.1). Three patients reported active smoking at the time of surgery. All individuals satisfied the institutional criteria regarding breast size and ptosis, specifically a nipple-to-sternal notch distance ≥ 26 cm and nipple-to-IMF distance ≥ 8 cm.

Surgical procedures involved 11 nipple-sparing mastectomies (NSM) and 5 skin-sparing mastectomies (SSM), for a total of 21 reconstructed breasts. Reconstructions were performed using:

- Submuscular techniques in six patients (37.5%), where flap thickness was ≤1 cm;
- Prepectoral reconstruction with ADM [20] in eight patients (50%), in cases where the pectoral fascia was not preserved;
- Prepectoral reconstruction with autologous fascial flaps in two patients (12.5%), with flap thickness and fascial integrity permitted.

Tumor staging ranged from pT1 to pT3, and three patients had prior breast surgeries. Six patients had comorbidities such as type II diabetes or hypertension. Breast composition was qualitatively assessed intraoperatively; 11 cases were predominantly adipose, and 5 were glandular-dense.

No major postoperative complications (e.g., implant loss, infections, or reoperations) were observed during the 3-month follow-up. Two cases of minor mastectomy flap necrosis (9.5%) occurred and were managed conservatively with local wound care, without impacting implant integrity or requiring surgical revision (Table 1).

The mobile application was employed during preoperative and intraoperative planning in all procedures. The app facilitated real-time standardization of the decision-making process among all surgical operators, with no intraoperative protocol deviations reported. Additionally, the app was found to be a practical support tool by all attending surgeons, including those in training, suggesting its utility in both clinical and educational contexts. This promoted a consistent surgical workflow and efficient execution of the algorithm. The time taken to complete intraoperative decisions using the app was consistently under 90 s. No intraoperative delays were reported.

Aesthetic outcomes and patient-reported outcomes were not systematically assessed in this pilot phase and will be evaluated in a forthcoming phase-II study.

## 4. Discussion

Immediate breast reconstruction represents a crucial therapeutic option to improve the quality of life in patients undergoing mastectomy for breast cancer. Several studies have demonstrated that immediate reconstruction, especially when combined with skin-sparing or nipple-sparing mastectomy, not only leads to satisfactory aesthetic outcomes but also reduces the psychological impact of breast loss, facilitating emotional adaptation to the disease and enhancing patients’ self-esteem [1,21].

However, immediate breast reconstruction presents challenges, particularly in patients with large and ptotic breasts. In these cases, residual skin redundancy and the management of cutaneous flaps may negatively affect the final aesthetic result and increase the risk of complications. These anatomical features represent a technical challenge for the plastic surgeon, who must adopt tailored reconstructive strategies that reconcile oncological safety with the aesthetic and functional demands of the patient.

Several reconstructive approaches have been described in the literature to manage immediate reconstruction in this subset of patients, including hybrid techniques, the use of acellular dermal matrices [22] pre-pectoral or subpectoral implants, and customized oncoplastic procedures [23,24,25].

At our institution, we have developed and validated a structured decision-making algorithm to guide the choice of the most appropriate technique according to the clinical and morphological characteristics of each patient. This algorithm has proven to be a valuable tool for standardizing the decision-making process, improving the uniformity of outcomes, and optimizing the overall aesthetic result [7,23].

In recent years, technological advancements have significantly influenced clinical practice in plastic and reconstructive surgery. In particular, the integration of digital tools, such as smartphone and tablet applications, has transformed both clinical workflows and educational processes. Current applications offer functionalities ranging from preoperative planning and postoperative monitoring to 3D simulation and secure data sharing between colleagues [10].

The application developed to support our institutional algorithm extends beyond a mere mnemonic aid. It functions as a comprehensive Clinical Decision Support System (CDSS), enabling a structured and evidence-based therapeutic approach. The platform is designed to be dynamic and modular, providing numerous advantages in daily clinical practice [26].

The use of digital technologies to enhance surgical outcomes is now well established across various specialties. Intraoperative AI systems leveraging computer vision and machine learning are being developed to support tissue identification, automate the recognition of surgical steps, and assist with complex decision-making during procedures [27,28]. An international consensus panel has also highlighted the potential of these technologies to reduce unwarranted practice variation and promote global equity in surgical care [29].

Our mobile application aligns with this broader movement in digital surgery by offering a simplified yet structured platform for intraoperative decision-making. While it does not incorporate advanced AI or video-based analytics, it operationalizes a validated clinical algorithm in a user-friendly format. This pragmatic design may offer particular advantages for surgical education, reproducibility across teams, and implementation in settings with limited technological infrastructure.

Interestingly, in all 16 procedures, the algorithm provided clear-cut guidance with no instances of ambiguity or deviation from the recommended path. Surgeons reported that the binary decisions were consistently applicable, and no judgment calls were required outside of the app’s logic.

Feedback was collected via anonymous post-operative questionnaires, completed by all five surgeons, rating the app’s usability, clarity, and clinical utility. All responses rated the app positively, citing its role in improving intraoperative consistency and reducing decision-making stress.

One of the primary benefits of the application lies in patient selection. The structured guidance minimizes the risk of error and reduces variability between individual surgeons. All users, regardless of experience level, can consult the algorithm and base their choices on objective clinical data, streamlining the evaluation process and enhancing efficiency.

Although the institutional algorithm informed current surgical culture, the app formalized this pathway and ensured adherence across all surgeons, particularly among residents who did not independently plan the reconstruction. This helped reduce intra-team variability.

Despite the growing integration of digital tools in surgical practice, validated decision-support systems in plastic and reconstructive surgery remain limited. Notably, no existing platform specifically addresses the intraoperative challenges of immediate breast reconstruction in large and ptotic breasts. This reinforces the innovative nature and potential impact of our proposed application.

Another key feature of the app is its educational utility, especially for residents and trainees. It supports the learning process by reinforcing protocol adherence and fostering the development of decision-making skills based on established guidelines and clinical pathways [30].

Although no structured evaluation of training outcomes was conducted, informal feedback suggested that residents found the app helpful in understanding algorithmic logic and surgical planning. Future versions will incorporate metrics such as decision accuracy and independent trainee assessments. Looking ahead, the application presents multiple opportunities for further development. For instance, expanding the internal database to include a wider range of reconstructive options and patient variables would enhance its flexibility and personalization capabilities. In addition, the platform can be regularly updated to reflect changes in national and international guidelines, ensuring that surgical decisions remain aligned with current best practices.

Lastly, it is essential to underscore that the application should not only be viewed as a decision-making tool but also as a platform for data collection, follow-up monitoring, and feedback acquisition. These functionalities are instrumental in refining the algorithm over time and improving the global management of patients undergoing mastectomy with immediate breast reconstruction.

## 5. Limitations

Despite the encouraging results of this preliminary study, several limitations must be acknowledged. First, the mobile application has, to date, been tested in a relatively small patient cohort from a single institution without randomization or a control group, which limits the generalizability of the findings. Additionally, the short-term follow-up does not allow for an adequate assessment of long-term outcomes.

Patient enrollment occurred between October 2023 and December 2024. Given the recent completion of recruitment, the average follow-up was 3 months, which limits long-term outcome analysis and is acknowledged in the limitations.

The decision algorithm reflects institutional practice and may not account for variations in surgical thresholds or the availability of materials such as ADM or fascial flaps in other centers. Future multicenter studies are warranted to assess external validity.

A further limitation lies in the technical scope of the app: the current version was developed exclusively for Android devices, potentially restricting its accessibility among users operating on iOS platforms. Future developments should aim to address these technical aspects, extend platform compatibility, and explore interoperability with surgical planning software and patient data registries.

The absence of a control group, formal usability metrics, and pre-post comparisons limits our ability to assess relative performance, learn curve effects, or make improvements in decision accuracy. Future iterations of this project will incorporate these elements.

## 6. Conclusions

The integration of a mobile application into the intraoperative decision-making process for immediate breast reconstruction represents a significant advancement in the surgical management of patients with large and ptotic breasts. In this anatomically and technically challenging subset, the use of a structured, algorithm-based digital tool facilitates standardization of reconstructive planning, reduces inter-operator variability, and supports evidence-based surgical strategies.

Our preliminary results demonstrate the clinical feasibility and utility of the app, which was successfully implemented in all cases without protocol deviations. The application proved to be a practical support tool for both experienced surgeons and trainees, reinforcing its dual function as a clinical decision aid and an educational resource.

In conclusion, this pilot study highlights the potential of digital decision-support tools to improve the quality and consistency of breast reconstruction practices. As technology continues to evolve, such innovations may play a pivotal role in advancing personalized care, optimizing surgical workflows, and enhancing training in plastic and reconstructive surgery.

## Figures and Tables

**Table 1:** Demographics, clinical data, and surgical type.

	AGE	BMI	SMOKE	BREAST WIDTH (LEFT)	NOCH TO NIPPLE (LEFT)	NIPPLE-IMF (LEFT)	BREAST WIDTH (RIGHT)	NOCH TO NIPPLE (RIGHT)	NIPPLE-IMF (RIGHT)	TYPE OF MASTECTOMY	TYPE OF RECONSTRUCTION	COMPLICATIONS
1	51	21.5	NO	17	26	8.5	17	26	9	SSM (RIGHT) NSM (LEFT)	SUBMUSCOLAR	NONE
2	52	31.7	NO	16	26	9	16	27	8	SSM (RIGHT)	PREPECTORAL WITH ADM	NONE
3	61	36.5	NO	17.5	27	9	17	26	8	NSM (BILATERAL)	PREPECTORAL WITH ADM	NONE
4	54	23.6	YES	12	28	8	11	28	8	NSM (BILATERAL)	PREPECTORAL WITH ADM	NONE
5	55	22	NO	12	26	8	12	26	9	NSM (RIGHT)	PREPECTORAL WITH ADM	NONE
6	49	25.7	NO	13	26	8.5	14	26	8.5	NSM(LEFT)	PREPECTORAL WITH ADM	NONE
7	47	19.72	NO	12.5	29	9.5	12.5	29	10	NSM (LEFT)	PREPECTORAL WITH ADM	NONE
8	59	21	YES	17	26	9	17	26	9	NSM (RIGHT)	SUBMUSCOLAR	NONE
9	68	27	NO	10	26.5	9	10	26.5	8.5	NSM (RIGHT)	SUBMUSCOLAR	NONE
10	55	28.3	NO	15	30	12	15	29	12	SSM LEFT	PREPECTORAL WITH ADM	NIPPLE NECROSIS
11	58	38.1	NO	17.5	26	16	17	26	18	NSM (RIGHT)	SUBMUSCOLAR IMPLANT	NONE
12	65	24.1	NO	11	27	8.5	12	28	9	SSM (RIGHT)	PREPECTORAL WITH FASCIAL FLAPS	NONE
13	61	32.4	YES	16.5	32	9	16	28	11.5	NSM (RIGHT)	SUBMUSCOLAR	MINOR SKIN NECROSIS
14	55	30	NO	15	30.5	8.5	14	28.5	11.5	NSM (LEFT)	PREPECTORAL WITH ADM	NONE
15	59	32.4	NO	16	28	9.5	16	27.5	11.5	NSM (LEFT)	PREPECTORAL WITH ADM	NONE
16	63	28	NO	15	28	9	15	26	9	NSM (RIGHT)	PREPECTORAL WITH FASCIAL FLAPS	NONE

SSM: skin sparing mastectomy, NSM: nipple sparing mastectomy, and ADM: acellular dermal matrix.

## Data Availability

The original contributions presented in this study are included in the article. Further inquiries can be directed to the corresponding author(s).
